# Non-Targeted Metabolomic Analyses Provide Insights into Exogenous Trehalose-Mediated Heat Stress Tolerance in Tea Plants (*Camellia sinensis* L.)

**DOI:** 10.3390/plants15131938

**Published:** 2026-06-23

**Authors:** Xiaohui Chen, Ziwei Zhou, Fang Wang, Chufei Liu, Rongzhao Lin, Shizhong Zheng

**Affiliations:** 1 College of Biological Science and Engineering, Ningde Normal University, Ningde 352100, China; 18950589675@163.com (X.C.); zwchow92@126.com (Z.Z.); fangcaopianpian@163.com (F.W.); chufei0626@126.com (C.L.); xuebizhenyin@gmail.com (R.L.); 2 College of Life Sciences, Fujian Normal University, Fuzhou 350117, China

**Keywords:** tea plant (*Camellia sinensis* L.), heat stress, exogenous trehalose, metabolites

## Abstract

Global warming exacerbates high-temperature stress, disturbing the growth, metabolic homeostasis and quality formation of tea plants (*Camellia sinensis* L.). Trehalose, a multifunctional osmolyte, can enhance abiotic stress tolerance, but its systematic metabolic mechanism against heat damage in tea remains unclear. Here, we applied integrated gas chromatography–mass spectrometry (GC-MS) and liquid chromatography–mass spectrometry (LC-MS) non-targeted metabolomics to compare control (CK), heat-stressed (T), and trehalose-treated heat-stressed (TT) tea leaves. We identified 163 differential volatile metabolites in GC-MS and 1619 differential non-volatile metabolites in LC-MS. Metabolite classification showed that organic oxygen compounds dominated differential volatile metabolites, while lipids and lipid-like molecules dominated differential non-volatile metabolites. The Kyoto Encyclopedia of Genes and Genomes enrichment showed that alanine, aspartate and glutamate metabolism, arginine biosynthesis, aminoacyl-tRNA biosynthesis, and flavone and flavonol biosynthesis were core shared pathways. Quantitatively, exogenous trehalose under heat stress significantly increased carbohydrate accumulation, restored lipid homeostasis, and elevated alanine, arginine, and related intermediates, thereby maintaining carbon–nitrogen balance. Trehalose also remodeled the amino acid substrate pool for aminoacyl-tRNA biosynthesis. In flavonoid metabolism, trehalose enhanced high-antioxidant flavonoid aglycones while reducing most glycosides and inhibiting excessive hydroxylation of flavonols. Although total flavonoid content decreased in TT relative to T, this reflected alleviated oxidative damage and reduced dependence on flavonoid-based defense. Combined with total amino acid and flavonoid quantifications, we conclude that exogenous trehalose enhances tea plant thermotolerance by coordinately regulating primary amino acid metabolism and secondary flavonoid metabolism. These findings provide a theoretical basis for using trehalose in heat-resistance cultivation and quality improvement of tea plants.

## 1. Introduction

Tea plant (*Camellia sinensis* L.) is an economically important perennial woody crop widely cultivated worldwide [1]. The leaves are rich in diverse metabolites and bioactive compounds, including amino acids, saccharides, flavonoids, organic acids, and alkaloids, which collectively determine the unique flavor, taste, and nutritional quality of tea products [2,3]. Tea plant growth relies heavily on stable temperature and precipitation, making it highly sensitive to climate change [4]. Consequently, climate change exerts extensive impacts on tea production, not only affecting the major tea-growing regions but also directly impairing tea yield and quality [4,5]. Moreover, extreme weather events caused by climate change, such as cold, heat, and drought, pose severe threats to the normal growth and development of tea plants [6,7,8]. Among these threats, heat stress has become particularly prominent with the intensification of global warming [9]. In southern China, tea plantations are frequently exposed to prolonged high temperatures and intense sunlight in summer, which severely disrupt plant growth and metabolic homeostasis [10]. High temperature stress impairs the C3 photosynthetic apparatus, reduces stomatal conductance and respiratory efficiency, and causes leaf scorching and metabolic disorders [11,12]. In terms of biochemical composition, heat stress typically elevates catechin and caffeine contents while decreasing amino acids, soluble sugars, and volatile aroma compounds in tea leaves, leading to strong bitterness, astringency, and inferior aroma of summer tea [13]. This quality decline results in significant waste of tea resources and restricts the economic benefits of the tea industry. Therefore, developing efficient and eco-friendly strategies to alleviate heat stress damage and improve tea quality is of great theoretical and practical importance.

Trehalose (α-D-glucopyranosyl-α-D-glucopyranoside) is a non-reducing disaccharide that functions as a vital stress protectant in diverse organisms [14]. As a multifunctional osmoprotectant and metabolic regulator, trehalose stabilizes cell membranes, proteins, and enzymes to alleviate thermal denaturation, remodels metabolic networks, maintains cellular turgor and water homeostasis [14,15], scavenges excessive reactive oxygen species (ROS) [16,17], and mediates stress signal transduction [18]. Accumulating studies have revealed that exogenous trehalose application or genetic modification of trehalose metabolism induces widespread reprogramming of primary and secondary metabolism under temperature stress [19,20]. Trehalose treatment significantly increases the accumulation of soluble sugars (e.g., glucose, sucrose, fructose) and sugar alcohols, which serve as osmoprotectants and energy sources, while also stabilizing carbohydrate metabolism-related enzymes and promoting carbon partitioning [21,22]. Trehalose alters levels of amino acids such as proline, alanine, arginine, and glutamate, helping to maintain carbon–nitrogen balance and providing precursors for protein synthesis under stress [23,24]. It also enhances the accumulation of flavonoids, phenolic compounds, and ascorbate–glutathione cycle intermediates, thereby reducing oxidative damage caused by heat or cold stress [25,26]. In addition, trehalose restores lipid homeostasis under heat stress, protecting membrane integrity [27]. For example, in *Ginkgo biloba*, foliar trehalose application has been shown to enhance thermotolerance by promoting phenylpropanoid, flavonoid, and terpenoid metabolism, strengthening cell wall integrity, elevating antioxidant enzyme activities, and reprogramming energy metabolism toward stress defense [28]. In wheat (*Triticum aestivum* L.), exogenous trehalose improves the physiological state of seedlings under heat stress by regulating carbohydrate metabolism, strengthening the tricarboxylic acid (TCA) cycle, and modulating the accumulation of protective metabolites including amino acids, purines, phenylpropanoids and flavonoids, while also regulating related gene expression [21]. In summary, trehalose acts as a regulator of metabolic homeostasis, orchestrating carbohydrate, amino acid, and secondary metabolite networks to enhance temperature stress tolerance in plants [29]. In tea plants, recent studies have shown that exogenous trehalose significantly mitigates heat-induced injuries by regulating phenotypic and physiological indices (e.g., increasing superoxide dismutase (SOD, EC1.15.1.1), peroxidase (POD, EC 1.11.1.7) activities, proline, endogenous trehalose and soluble sugar contents, while reducing malondialdehyde (MDA), hydrogen peroxide (H_2_O_2_) and superoxide radical (O_2_^−^) levels), upregulating stress-responsive genes (e.g., *HSFB2B*, *HSP18.1* and *HSP26.5*) involved in the mitogen-activated protein kinase pathway and phenylpropanoid and flavonoid biosynthesis, and modulating circular RNA (circRNA) regulatory profiles, including differential expression circRNAs and their host genes related to DNA double-strand break repair pathways [30,31]. However, the global metabolic changes and systematic regulatory network underlying trehalose-mediated thermotolerance in tea plants remain largely unclear.

Metabolomics is a powerful tool for comprehensively profiling low-molecular-weight metabolites and revealing dynamic metabolic adaptations in plants under stress [32]. Changes in metabolite profiles directly reflect physiological status and metabolic regulation in response to adverse environments [33]. Among various analytical platforms, gas chromatography–mass spectrometry (GC-MS) and liquid chromatography–mass spectrometry (LC-MS) are the most widely used techniques due to their high sensitivity, resolution, and throughput, which allow the simultaneous identification and quantification of hundreds of metabolites. Specifically, GC-MS is suitable for characterizing volatile and thermostable compounds, whereas LC-MS excels in analyzing non-volatile, heat-labile, and high-molecular-weight substances that are beyond the detection scope of GC-MS [34,35]. In the present study, a comprehensive metabolomics approach integrating GC-MS and LC-MS was applied, accompanied by the quantification of total amino acids and total flavonoids, to systematically characterize metabolic alterations in tea plants under control (CK), heat stress (T), and heat stress with exogenous trehalose application (TT) conditions. This study aims to identify key differential metabolites and metabolic pathways affected by heat stress and trehalose treatment, to reveal the regulatory effects of trehalose on metabolism, and to clarify the metabolic mechanism by which exogenous trehalose enhances thermotolerance in tea plants. The findings will provide new insights into the molecular basis of trehalose-mediated stress tolerance and lay a theoretical foundation for the application of trehalose in heat-resistance cultivation of tea plants.

## 2. Results

### 2.1. Quality Control and Multivariate Statistical Analysis of GC-MS Metabolomic Profiling

**Figure 1:**
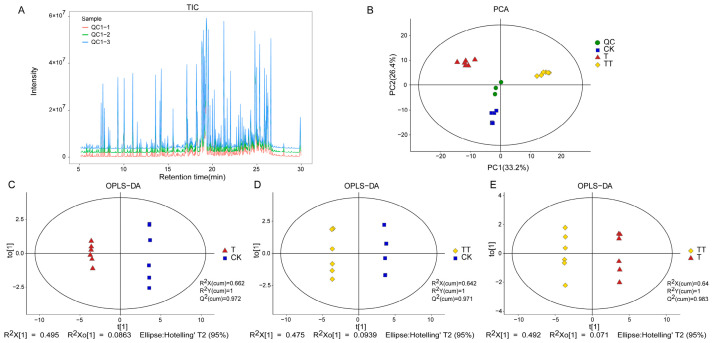
GC-MS-based quality control and multivariate statistical analysis of volatile metabolites. (**A**) Total ion chromatography (TIC) overlay of all quality control (QC) samples showed extensive peak overlap in both retention times and response intensities, indicating minimal systematic error during the analysis. (**B**) The PCA score plot demonstrated high intragroup cohesion and clear intergroup separation among the three groups (CK, T, and TT), reflecting distinct differences in their volatile metabolite profiles. (**C**–**E**) Orthogonal partial least squares discriminant analysis (OPLS-DA) score plots for the pairwise comparisons (T vs. CK, TT vs. CK, and TT vs. T) with 95% Hotelling T^2^ ellipse. R^2^X (cum) represents the cumulative explained variance of the model in the X-direction during multivariate statistical analysis modeling, where “cum” denotes the cumulative result of several principal components; R^2^Y (cum) represents the cumulative explained variance of the model in the Y-direction; and Q^2^ (cum) represents the cumulative predictive ability of the model.

To comprehensively characterize the metabolite landscape of the three experimental groups and verify the reliability of the analytical workflow, quality control assessment combined with multivariate statistical analysis was conducted in GC-MS. First, complementary total ion chromatography (TIC) curve analysis was performed to evaluate the stability of the analytical system. As shown in Figure 1A, extensive overlap in peak response intensities and retention times across samples was observed, suggesting that instrumental variation was minimal throughout the experiment. Subsequently, principal component analysis (PCA) was performed to intuitively assess overall differences in metabolite profiles between the groups. As presented in Figure 1B, the coordinate points of the three groups were well-separated in the PCA score plot, indicating distinct metabolite profile divergence among them; meanwhile, quality control (QC) samples clustered tightly near the center of all samples, confirming good experimental reproducibility. Together, these results further supported that the observed differences between groups originated from true metabolite-level variations, rather than system instability during data acquisition. To further enhance the accuracy of differential metabolite screening by focusing on targeted pairwise comparisons between groups, orthogonal partial least squares discriminant analysis (OPLS-DA) models were established for T vs. CK (R^2^X (cum) = 0.662, R^2^Y (cum) = 1, Q^2^ (cum) = 0.972; Figure 1C), TT vs. CK (R^2^X (cum) = 0.642, R^2^Y(cum) = 1, Q^2^ (cum) = 0.971; Figure 1D), and TT vs. T (R^2^X (cum) = 0.64, R^2^Y (cum) = 1, Q^2^ (cum) = 0.983; Figure 1E). Notably, the Q^2^ (cum) values of all three comparison groups were higher than 0.97, demonstrating that the OPLS-DA models possessed excellent predictive ability (Supplementary Table S1). Furthermore, 200 response permutation tests were conducted to assess potential overfitting of OPLS-DA models. The permuted Q^2^ values for T vs. CK, TT vs. CK and TT vs. T were −0.233, −0.137 and −0.306, respectively (Supplementary Figure S1). These negative Q^2^ values confirm that all models are robust and free from overfitting. Collectively, the reliable model performance provides a solid basis for subsequent screening of differential metabolites.

### 2.2. Quality Control and Multivariate Statistical Analysis of LC-MS Metabolomic Profiling

**Figure 2:**
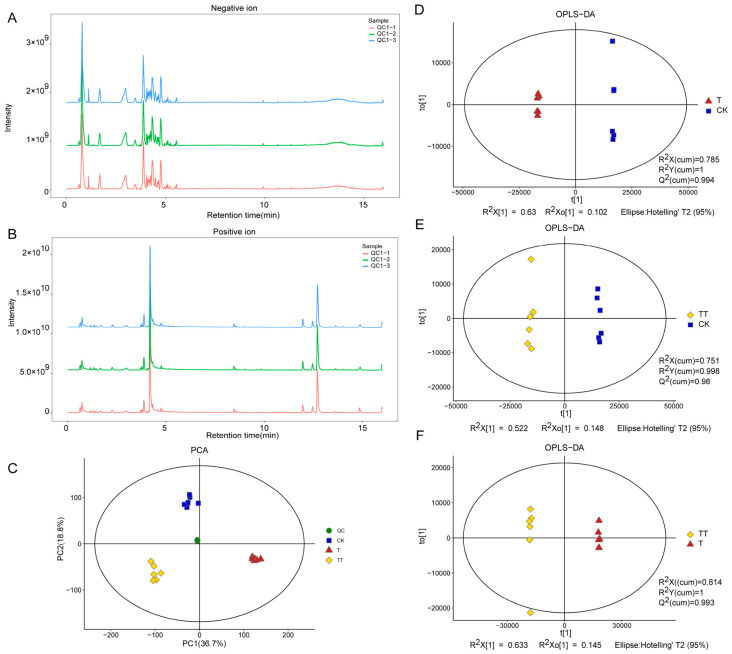
LC-MS quality control and analytical reliability assessment. (**A**,**B**) BPC of positive and negative ion modes. The intensities of the strongest ions at each time point were highly consistent and overlapped, indicating excellent stability of the LC-MS system. (**C**) PCA score plot showing high intra-group cohesion and clear inter-group separation among the three groups. (**D**–**F**) OPLS-DA score plots for the pairwise comparisons (T vs. CK, TT vs. CK, and TT vs. T) with 95% Hotelling T^2^ ellipse. R^2^X (cum) represents the cumulative explained variance of the model in the X-direction during multivariate statistical analysis modeling, where “cum” denotes the cumulative result of several principal components; R^2^Y (cum) represents the cumulative explained variance of the model in the Y-direction; and Q^2^ (cum) represents the cumulative predictive ability of the model.

Consistent with the GC-MS workflow, quality control assessment and multivariate statistical analysis were conducted for LC-MS as well. The base peak chromatogram (BPC) of both positive and negative ion modes showed that the intensities of the strongest ions at each time point were highly consistent and overlapped, demonstrating robust instrument performance (Figure 2A,B). PCA was then performed on LC-MS samples from the three groups to evaluate overall metabolite profile differences. As shown in Figure 2C, the three groups exhibited clear separation on the PCA score plot, and replicates within each group clustered closely together. Meanwhile, QC samples clustered closely together, further verifying instrumental stability and the reproducibility and reliability of the test data. To further enhance the resolution of group-specific metabolic differences, OPLS-DA analysis was applied to the samples (Figure 2D–F, Supplementary Table S2). The results showed that all samples from the three groups were well separated, and the model parameters R^2^Y (cum) and Q^2^ (cum) scores were ≥0.9, confirming the validity of the observed metabolic differences among the three treatment groups. In addition, the permuted Q^2^ values were −0.403 (T vs. CK), −0.34 (TT vs. CK) and −0.286 (TT vs. T) (Supplementary Figure S2). The negative Q^2^ results demonstrate that all OPLS-DA models are robust without overfitting. Such reliable model performance ensures the accurate identification of differential metabolites in the following analyses.

### 2.3. GC-MS- and LC-MS-Based Metabolomics Analysis of Differential Volatile and Non-Volatile Metabolites in Tea Plants Under Heat Stress with Exogenous Trehalose Treatment

Given that multivariate analyses including PCA and OPLS-DA had confirmed significant metabolic differences among the CK, T, and TT samples, Student’s *t*-test and fold change (FC) analysis were further employed to compare metabolite profiles between each comparison group.

**Figure 3:**
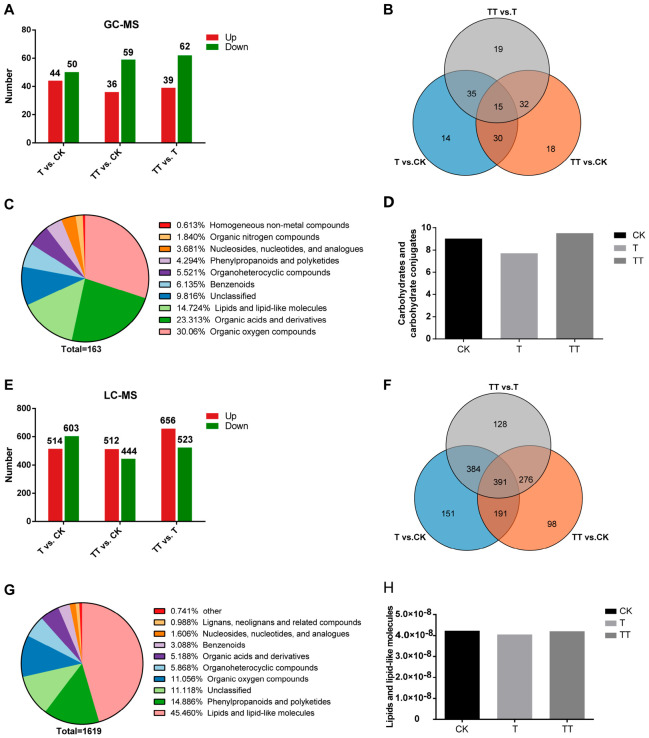
Statistical analysis of differential metabolites from GC-MS and LC-MS. (**A**–**D**) GC-MS differential volatile metabolites (DVMs): (**A**) number of DVMs in pairwise comparisons (T vs. CK, TT vs. CK, TT vs. T); (**B**) Venn diagram of overlapping DVMs across groups; (**C**) chemical classification of 163 DVMs; (**D**) contents of carbohydrates and carbohydrate conjugates in CK, T, and TT groups; (**E**–**H**) LC-MS differential non-volatile metabolites (DNMs): (**E**) number of DNMs in pairwise comparisons; (**F**) Venn diagram of overlapping DNMs across groups; (**G**) chemical classification of 1619 DNMs; (**H**) contents of lipids and lipid-like molecules in CK, T, and TT groups.

Through GC-MS analysis, a total of 163 differential volatile metabolites (DVMs) were screened from the database. Pairwise comparisons identified 94, 95, and 101 DVMs in the T vs. CK, TT vs. CK, and TT vs. T groups, respectively (Figure 3A–D). These 163 DVMs were further classified into 10 major classes (Figure 3C), among which organic oxygen compounds were the most dominant (30.06%), followed by organic acids and derivatives (23.31%), lipids and lipid-like molecules (14.72%), benzenoids (6.13%), organoheterocyclic compounds (5.52%), phenylpropanoids and polyketides (4.29%), nucleosides, nucleotides, and analogues (3.68%), organic nitrogen compounds (1.84%), homogeneous non-metal compounds (0.61%), and unclassified metabolites (9.82%). These organic oxygen compounds (49 metabolites, 30.06% of total DVMs) were further classified into three major subclasses: carbohydrates and carbohydrate conjugates (mainly monosaccharides, di-/tri-/oligosaccharides, phosphorylated sugars, sugar acids, amino sugars, glycosides, and other derivatives), alcohols and polyols, and carbonyl compounds. Among these, carbohydrates and carbohydrate conjugates constituted the predominant subclass. Further analysis of their relative contents revealed that the contents of carbohydrates and carbohydrate conjugates were increased in both the T and TT groups, with an even higher level of accumulation observed in the TT group (Figure 3D and Supplementary Table S3). This result suggests that heat stress significantly reshaped the overall metabolic profile of organic oxygen compounds in tea plants, and that trehalose may mitigate heat stress by enhancing the accumulation of sugars and related osmoprotectants.

For LC-MS analysis, a total of 1619 differential non-volatile metabolites (DNMs) were identified. Among these, 1117, 956, and 1179 DNMs were detected in T vs. CK, TT vs. CK, and TT vs. T, respectively (Figure 3E–H). The 1619 DNMs were classified into 10 categories, with the top eight being lipids and lipid-like molecules (45.46%), phenylpropanoids and polyketides (14.89%), organic oxygen compounds (11.12%), organoheterocyclic compounds (5.87%), organic acids and derivatives (5.19%), benzenoids (3.09%), nucleosides, nucleotides, and analogues (1.61%), and lignans, neolignans and related compounds (0.99%). The remaining metabolites were assigned to “other” (0.74%) and “unclassified” (11.12%) (Figure 3F). A total of 736 lipids and lipid-like molecules were further classified into eight subclasses: fatty acyls, glycerolipids, glycerophospholipids, polyketides, prenol lipids, saccharolipids, sphingolipids, and steroids and steroid derivatives (including sterol lipids). Compared with the CK group, the content of lipids and lipid-like molecules was downregulated under heat stress, but restored to near-CK levels in the trehalose treatment group, indicating that trehalose may alleviate the heat-induced inhibition of lipid biosynthesis and stimulate its synthesis (Figure 3H and Supplementary Table S4).

### 2.4. KEGG Pathway Functional Enrichment Analyses of Differential Metabolites

To further investigate the molecular basis underlying secondary metabolic differences, KEGG pathway enrichment analysis was performed on the identified differential metabolites.

**Figure 4:**
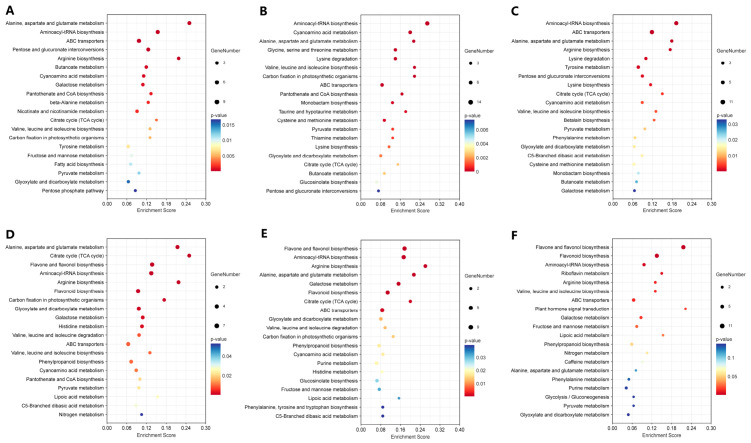
KEGG enrichment analysis of the differentially expressed volatile/non-volatile metabolites identified in T vs. CK, TT vs. CK, and TT vs. T, respectively. (**A**) DVMs in T vs. CK. (**B**) DVMs in TT vs. CK. (**C**) DVMs in TT vs. T. (**D**) DNMs in T vs. CK. (**E**) DNMs in TT vs. CK. (**F**) DNMs in TT vs. T.

As shown in Figure 4A–C, distinct KEGG enrichment patterns were observed for DVMs across the three comparison groups. In T vs. CK, the significantly enriched metabolic pathways included alanine, aspartate and glutamate metabolism, aminoacyl-tRNA biosynthesis, ABC transporter, pentose and glucuronate interconversions, arginine biosynthesis, butanoate metabolism, and cyanoamino acid metabolism (Figure 4A). For TT vs. CK, the enriched pathways were concentrated in aminoacyl-tRNA biosynthesis, cyanoamino acid metabolism, alanine, aspartate and glutamate metabolism, and glycine, serine and threonine metabolism (Figure 4B). Meanwhile, in TT vs. T, the top five significantly enriched pathways were aminoacyl-tRNA biosynthesis, ABC transporter, alanine, aspartate and glutamate metabolism, arginine biosynthesis, and lysine degradation (Figure 4C).

Similarly, the KEGG enrichment results for DNMs (Figure 4D–F) revealed pathway-specific changes associated with exogenous trehalose treatment under heat stress. In T vs. CK, the significantly enriched metabolic pathways included alanine, aspartate and glutamate metabolism, TCA cycle, flavone and flavonol biosynthesis, aminoacyl-tRNA biosynthesis, arginine biosynthesis, and flavonoid biosynthesis (Figure 4D). For TT vs. CK, the enriched pathways expanded to include flavone and flavonol biosynthesis, aminoacyl-tRNA biosynthesis, arginine biosynthesis, alanine, aspartate and glutamate metabolism, galactose metabolism, flavonoid biosynthesis, TCA cycle, and ABC transporter (Figure 4E). In TT vs. T, the significantly enriched pathways were dominated by flavone and flavonol biosynthesis, flavonoid biosynthesis, aminoacyl-tRNA biosynthesis, galactose metabolism, ABC transporter, and arginine biosynthesis (Figure 4F).

It is worthy of note that both alanine, aspartate and glutamate metabolism and aminoacyl-tRNA biosynthesis are common pathways in the DVMs across the three comparison groups. In contrast, arginine biosynthesis, aminoacyl-tRNA biosynthesis, and flavone and flavonol biosynthesis were identified as shared pathways in the DNMs among the T vs. CK, TT vs. CK and TT vs. T groups. These results suggested that the above pathways were actively involved in the metabolic regulation of tea plants under heat stress with exogenous trehalose treatment.

### 2.5. Analysis of DVMs Involved in Alanine, Aspartate and Glutamate Metabolism and DNMs Involved in Arginine Biosynthesis in Tea Plants Under Heat Stress with Exogenous Trehalose Treatment

**Figure 5:**
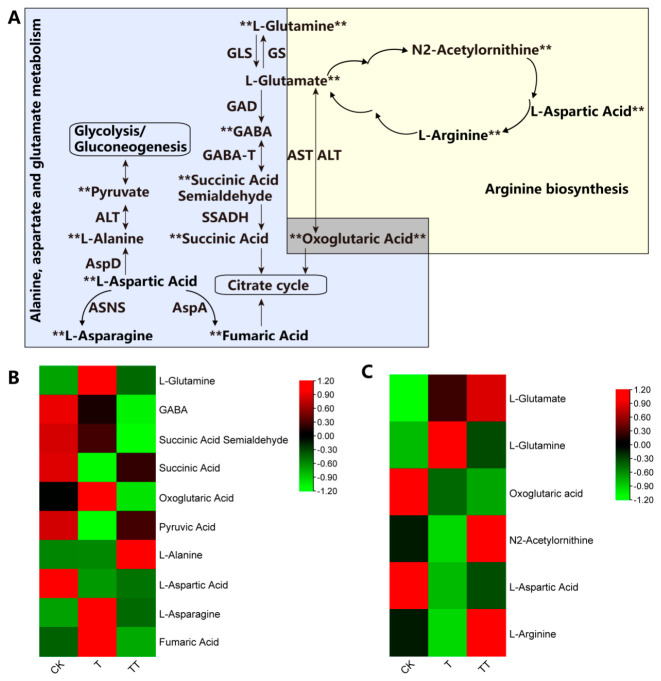
Analysis of DVMs involved in alanine, aspartate, and glutamate metabolism and DNMs involved in arginine biosynthesis pathway. (**A**) Schematic diagram of alanine, aspartate, and glutamate metabolism and arginine biosynthesis. (**B**) Heatmap showing the DVMs in the CK, T and TT groups. (**C**) Heatmap showing the DNMs in the CK, T and TT groups. The asterisks (**) on the left denote DVMs involved in alanine, aspartate, and glutamate metabolism. The asterisks (**) on the right represents DNMs involved in the arginine biosynthesis pathway. ALT: alanine aminotransferase; AspD: L-aspartate 4-decarboxylase; ASNS: asparagine synthetase; AspA: aspartate ammonia-lyase; GS: glutamine synthetase; GLS: glutaminase; GAD: glutamate decarboxylase; GABA-T: 4-aminobutyrate aminotransferase; SSADH: succinate-semialdehyde dehydrogenase.

As key branches of amino acid metabolism, the alanine, aspartate and glutamate metabolism pathway and the arginine biosynthesis pathway are interconnected via intermediate metabolites (e.g., α-ketoglutarate, glutamate, aspartate, etc.) and jointly feed into the TCA cycle. Based on these identified differential metabolites, a pathway map was constructed to intuitively illustrate their metabolic involvement in these two pathways (Figure 5A).

A total of 10 DVMs were identified within the alanine, aspartate, and glutamate metabolism pathway, including L-glutamine, GABA, succinic acid semialdehyde, succinic acid, oxoglutaric acid, pyruvic acid, L-alanine, L-aspartic acid, L-asparagine, and fumaric acid. Comparative analysis of their abundances across the CK, T, and TT groups revealed a consistent decrease in GABA, succinic acid semialdehyde, succinic acid, and L-aspartic acid in both T and TT samples relative to CK. This pattern may indicate a significant inhibition of the TCA cycle in tea plants under heat stress. Notably, in the TT group, L-alanine was significantly increased, and the pyruvic acid content was higher compared to the T group (Figure 5B). Alanine not only participates in nitrogen metabolism but also acts as an important energy substrate under stress, especially when carbon supply is restricted [36]. These results indicated that exogenous trehalose supplementation could enhance glycolytic flux, providing sufficient carbon skeletons to support downstream stress-responsive metabolic synthesis.

Furthermore, six DNMs were annotated to the arginine biosynthesis pathway, namely L-glutamine, L-glutamate, oxoglutaric acid, N2-acetylornithine, L-aspartic acid, and L-arginine. Notably, the concentrations of L-glutamate, N2-acetylornithine, and L-arginine were significantly upregulated in the TT group (Figure 5C). Arginine acts as an important precursor for signaling molecules such as nitric oxide (NO) and for polyamines including putrescine, spermidine, and spermine, in addition to functioning as an osmoprotectant [37]. These results suggest that trehalose treatment enhances cellular signaling and stress resistance in tea plants under heat stress, potentially through the promotion of arginine biosynthesis.

### 2.6. Analysis of DVMs and DNMs Involved in Aminoacyl-tRNA Biosynthesis in Tea Plants Under Heat Stress with Exogenous Trehalose Treatment

**Figure 6:**
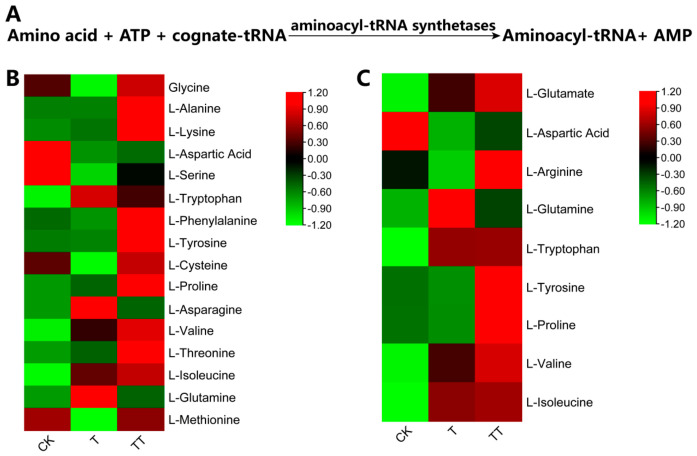
Analysis of DVMs and DNMs involved in aminoacyl-tRNA biosynthesis. (**A**) The core catalytic reaction of aminoacyl-tRNA biosynthesis. (**B**) Heatmap showing the DVMs in the CK, T and TT groups. (**C**) Heatmap showing the DNMs in the CK, T and TT groups.

Aminoacyl-tRNA biosynthesis is an ATP-dependent process and a key step in protein synthesis catalyzed by aminoacyl-tRNA synthetases. Its core function is to ensure the accurate decoding of the genetic code: each aminoacyl-tRNA synthetase specifically recognizes one amino acid and its corresponding tRNA, charges the tRNA with the correct amino acid, and delivers it to the ribosome for polypeptide chain elongation, thereby guaranteeing the fidelity of protein synthesis (Figure 6A). This pathway acts as a critical bridge for the transfer of genetic information from mRNA to protein and was significantly modulated by heat stress and exogenous trehalose treatment in tea plants. Among the 16 DVMs associated with this pathway, 10 amino acids (glycine, L-alanine, L-lysine, L-phenylalanine, L-tyrosine, L-cysteine, L-proline, L-valine, L-threonine, and L-isoleucine) showed increased relative levels in TT samples compared to T samples (Figure 6B). Similarly, within the nine DNMs mapped to the same pathway, seven amino acids (L-glutamate, L-arginine, L-tryptophan, L-tyrosine, L-proline, L-valine, and L-isoleucine) also exhibited upward trends in the TT group (Figure 6C). These findings suggested that exogenous trehalose application under heat stress reshapes the amino acid substrate pool for aminoacyl-tRNA biosynthesis, which may indicate a re-prioritization of protein synthesis or an adaptive translational response in tea plants under heat stress.

### 2.7. Analysis of DNMs and DEGs Involved in Flavone and Flavonol Biosynthesis in Tea Plants Under Heat Stress with Exogenous Trehalose Treatment

**Figure 7:**
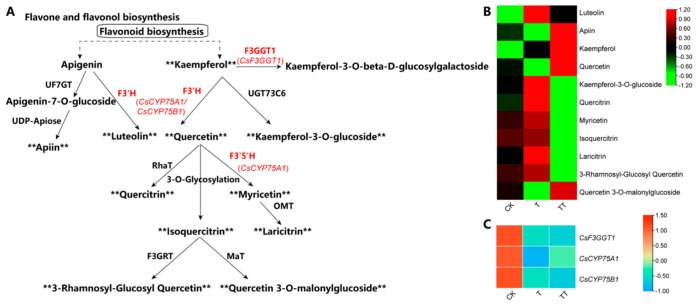
Analysis of DNMs involved in flavone and flavonol biosynthesis. (**A**) Metabolic pathway map of flavone and flavonol biosynthesis. (**B**) Heatmap showing the DNMs in the CK, T and TT groups. The asterisks (**) indicate DNMs in flavone and flavonol biosynthesis. (**C**) Heatmap showing the DEGs in the CK, T and TT groups. UF7GT: UDP-glucose:flavanone 7-O-glucosyltransferase; F3′H: flavonoid 3′-hydroxylase; UGT73C6: flavonol-3-O-L-rhamnoside-7-O-glucosyltransferase; RhaT: UDP-rhamnose:quercetin 3-O-rhamnosyltransferase; F3′5′H: flavonoid 3′,5′-hydroxylase; OMT: myricetin 3′-O-methyltransferase; F3GRT: flavonol-3-O-glucoside L-rhamnosyltransferase; MaT: malonyl-CoA: flavonoid-O-glucoside malonyltransferase.

A total of 11 DNMs were identified in the flavone and flavonol biosynthesis pathway (Figure 7A). Among them, aglycones including kaempferol and quercetin showed significantly increased accumulation in the TT group, whereas major glycoside derivatives such as kaempferol-3-O-glucoside, quercitrin, isoquercitrin, and 3-rhamnosyl-glucosyl quercetin were generally downregulated. This indicated that TT treatment enhanced the accumulation of flavonol aglycones while reducing their corresponding glycosylated products. Notably, despite the overall downregulation of most glycosides, quercetin 3-O-malonylglucoside displayed an upward trend. Moreover, the contents of polyhydroxylated flavonols including myricetin and laricitrin were decreased, implying that the hydroxylation conversion from quercetin to highly hydroxylated flavonols was inhibited under heat stress with trehalose application (Figure 7B).

Transcriptome analysis revealed that differentially expressed genes (DEGs) were significantly enriched in the flavone and flavonol biosynthesis pathway, and three key structural genes were identified, namely *CsF3GT1* (*flavonoid 3-O-glucosyltransferase*), *CsCYP75A* (*flavonoid 3′,5′-hydroxylase 1*), and *CsCYP75B1* (*flavonoid 3′-monooxygenase*). Analysis of gene expression patterns showed that heat stress significantly repressed the transcription of *CsCYP75A1* and *CsCYP75B1*, thereby weakening the catalytic reactions from kaempferol to quercetin and further to myricetin (Figure 7C). Combined with metabolomic profiles, exogenous trehalose altered the metabolic flux of flavonols from the myricetin branch toward quercetin accumulation by differentially regulating the expression of *CsCYP75A1* and *CsCYP75B1* under heat stress. Collectively, these coordinated changes in metabolites and genes demonstrate the specific regulatory mechanism of trehalose in modulating flavonoid metabolism in tea plants during heat stress.

### 2.8. Response Characteristics of Amino Acids and Flavonoids in Tea Plants Under Heat Stress with Exogenous Trehalose Treatment

**Figure 8:**
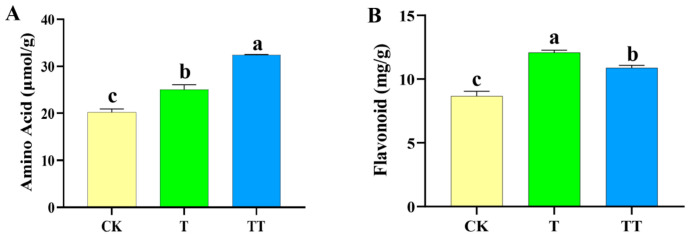
Analysis of key differential metabolites in CK, T, and TT groups. (**A**) Contents of amino acids in CK, T, and TT groups. (**B**) Contents of flavonoids in CK, T, and TT groups. Error bars represent mean ± SD (n = 3). Different lowercase letters indicate significant differences among treatments at *p* < 0.05.

DVMs and DNM analysis revealed that organic oxygen compounds and lipids and lipid-like molecules were the dominant differential metabolites in the CK, T, and TT groups. Among these, carbohydrates and carbohydrate conjugates (including monosaccharides, di-/tri-/oligosaccharides, phosphorylated sugars, sugar acids, amino sugars, glycosides, and other derivatives) as well as polyketides (mainly flavonoids) were prominent (Figure 3). Furthermore, KEGG enrichment analysis showed that alanine, aspartate and glutamate metabolism, arginine biosynthesis, aminoacyl-tRNA biosynthesis, and flavone and flavonol biosynthesis were significantly enriched (Figure 4), with amino acids and flavonoids as the key responsive components. Given that our previous study had determined soluble sugar contents under heat stress with trehalose treatment [30], we further quantified total amino acid and total flavonoid contents in the CK, T, and TT groups in the present study (Figure 8).

The results showed that the total amino acid content in the TT group was significantly higher than that in both the CK and T groups (Figure 8A). As important osmotic regulators and stress-protective substances in tea plants, the increased accumulation of amino acids may enhance the heat stress tolerance of tea plants. For flavonoids, the typical secondary stress-resistant metabolites in tea plants, the total flavonoid content in the TT group was significantly higher than that in the CK group but significantly lower than that in the T group (Figure 8B). This suggested that trehalose may directly alleviate heat stress damage to tea plants and significantly reduce the intensity of stress signals in tea plant cells, thus leading to a decrease in flavonoid content, which is a clear physiological sign that the heat stress of tea plants has been effectively mitigated.

## 3. Discussion

Exogenous trehalose, a crucial non-reducing sugar and effective osmoprotectant, has been widely acknowledged to alleviate the detrimental impacts of various abiotic stresses in plants [18,29,38]. It functions by regulating cellular metabolic processes, sustaining cellular and structural integrity, enhancing the antioxidant defense system, and activating endogenous stress-related molecular and physiological responses [20,39]. Our research group has previously investigated the morphological changes, gene expression patterns, and circRNA regulatory profiles of tea plants in response to trehalose application under heat stress, which has provided important clues for elucidating the regulatory mechanisms of trehalose in alleviating heat stress damage in tea plants [30,31]. However, the metabolic changes induced by exogenous trehalose in tea plants under heat stress remain largely unreported. In this study, an integrated metabolomic approach combining GC-MS and LC-MS with targeted metabolite content determination was employed to systematically elucidate the metabolic regulatory mechanisms underlying trehalose-mediated heat stress tolerance in tea plants.

### 3.1. Trehalose Modulates the Accumulation of Carbohydrates and Carbohydrate Conjugates, and Lipids Molecules to Enhance Heat Tolerance in Tea Plants

Multivariate statistical analysis of GC-MS and LC-MS data confirmed the reliability of the metabolomic profiling workflow and significant metabolic divergence among CK, T, and TT groups (Figure 1 and Figure 2). A total of 163 DVMs and 1619 DNMs were identified, with organooxygen compounds and lipids and lipid-like molecules being the most abundant classes of DVMs and DNMs, respectively (Figure 3).

Within the organooxygen compounds, carbohydrates and their conjugates represented the predominant subclass. These metabolites perform diverse physiological functions, such as carbon storage and transport, and also play essential roles in stress tolerance. For instance, in *Arabidopsis*, the levels of primary carbohydrates including sucrose, raffinose, and maltose increase following exposure to 40 °C for up to 4 h [40]. Additionally, during the first 24 h of heat stress, 15% of upregulated proteins in soybean are associated with carbohydrate metabolism [40]. Similarly, in developing pea (*Pisum sativum* L., cv Baccara) seeds, the accumulation of raffinose and stachyose, together with an elevated (raffinose + stachyose)/sucrose ratio, is closely associated with the acquisition of desiccation tolerance, as oligosaccharides likely protect membranes during dehydration [41]. In tea plants, exogenous trehalose has been shown to significantly increase soluble sugar contents under high-temperature conditions [30]. Consistent with these findings, we observed a substantial accumulation of carbohydrates and carbohydrate conjugates in trehalose-treated tea plants. Furthermore, KEGG enrichment analysis showed that galactose metabolism was notably enriched in the TT vs. T group comparison, suggesting an association between trehalose treatment and altered metabolic flux of galactose-related pathways. Collectively, these observations indicate that trehalose is associated with altered sugar metabolism and increased carbohydrate levels, which may help mitigate heat-induced damage.

Lipids are indispensable components in plants, contributing fundamentally to photosynthesis, membrane stability, antioxidative defense, protein glycosylation, growth and development, and stress tolerance [42]. Heat stress directly disrupts membrane fluidity and integrity by altering lipid composition and lipid–protein interactions, while excessive ROS accumulation further impairs membrane function and antioxidant enzyme activity [43,44]. To mitigate heat-induced damage, plants actively remodel membrane lipids by reducing fatty acid unsaturation, adjusting the ratio of lipid classes such as monogalactosyldiacylglycerol/digalactosyldiacylglycerol, and regulating the levels of phospholipids including phosphatidylcholine, phosphatidylethanolamine, and phosphatidylinositol [45,46]. Moreover, the heat-tolerant soybean genotype showed a low level of fatty acid desaturase expression due to the decreased level of unsaturated fatty acids under heat stress for maintaining membrane functionality [47]. In this study, lipids and lipid-like molecules were the most abundant category among all significantly changed metabolites. Their predominance indicated that trehalose preferentially modulates lipid metabolism in tea plants under heat stress. Notably, relative to the heat-stressed group (T), trehalose supplementation restored lipid accumulation in the TT group to levels comparable to those in the CK group. This suggested that trehalose is capable of maintaining membrane stability by regulating lipid metabolism, thereby enhancing the thermotolerance of tea plants under heat stress.

### 3.2. Trehalose-Mediated Changes in Amino Acid Metabolism, Protein Translation and Flavonoid Biosynthesis in Heat-Stressed Tea Plants

KEGG pathway enrichment analysis revealed that the DVMs were mainly enriched in alanine, aspartate and glutamate metabolism and aminoacyl-tRNA biosynthesis, while the DNMs were predominantly enriched in arginine biosynthesis, aminoacyl-tRNA biosynthesis, and flavone and flavonol biosynthesis across pairwise comparisons (Figure 4). Further KEGG analysis indicated clear interactions and overlapping relationships between the enriched pathways of DVMs and DNMs. Alanine, aspartate and glutamate metabolism serves as a critical hub connecting carbohydrate and amino acid metabolism, and arginine biosynthesis represents an important branch within plant amino acid networks. In addition, aminoacyl-tRNA biosynthesis was identified as a common shared pathway between DVMs and DNMs.

Under stress-induced energy deficiency, plants repurpose amino acids from protein building blocks into alternative respiratory substrates and signaling molecules [48]. For instance, proline and alanine accumulate to high levels under abiotic stress and stimulate respiration by transcriptionally upregulating their corresponding degradation pathways [48]. In addition, alanine serves as a pivotal intermediate linking glycolysis and nitrogen metabolism, thereby maintaining cellular carbon–nitrogen homeostasis [36]. Under hypoxia, plants reconfigure nitrogen metabolism via the coordinated regulation of ALT (Alanine aminotransferase) and GDH (glutamate dehydrogenase). ALT drives substantial alanine accumulation by catalyzing the conversion of pyruvate and glutamate, which facilitates safe carbon and nitrogen storage and avoids cytoplasmic acidification. Upon reoxygenation, accumulated alanine is reversibly degraded to replenish the TCA cycle, while GDH mediates nitrogen recycling to restore cellular metabolic homeostasis [49]. Consistent with this regulatory mechanism, the alanine, aspartate and glutamate metabolism pathway was significantly activated in this study, with alanine content markedly increased in the TT group. Collectively, these findings indicated that trehalose may enhance glycolytic flux to supply sufficient carbon skeletons for energy metabolism. This inference is further supported by the increased accumulation of carbohydrates and carbohydrate conjugates in trehalose-treated tea plants under heat stress.

Arginine metabolism plays a vital role in regulating plant stress tolerance. As an important metabolic precursor, arginine contributes to the synthesis of NO and polyamines, both of which participate in the regulation of biotic and abiotic stress responses as well as plant developmental processes [37]. For example, *SlNAGS1*-overexpressing *Arabidopsis* accumulated more leaf ornithine and exhibited stronger salt and drought tolerance than wild plants. The improved stress tolerance was closely linked to elevated ornithine, citrulline and arginine, which commonly accumulate with proline in salt-stressed higher plants [50,51]. Consistent with these findings, exogenous arginine enhances the growth and phycobiliprotein content of *Gracilariopsis lemaneiformis* under high temperature. It mitigates heat-induced oxidative damage by lowering MDA levels, boosting antioxidant enzyme (SOD, POD, catalase (CAT, EC 1.11.1.6)) activities and free amino acid accumulation, while transcriptionally upregulating antioxidant- and heat shock-related genes as well as transcription factors (TFs), and modulating arginine–proline metabolism, amino acid biosynthesis, glycolysis and the TCA cycle to restore cellular carbon and nitrogen homeostasis [52]. These results suggest that stress can induce the accumulation of amino acids such as proline and arginine in plant tissues, which in turn activates stress-responsive genes and metabolic enzymes to enhance stress adaptability. In our study, the concentrations of L-glutamate, N2-acetylornithine, and L-arginine were significantly upregulated in the TT group, indicating that trehalose treatment induces arginine accumulation in tea plants under heat stress, thereby strengthening cellular signal transduction and stress resistance.

Aminoacyl-tRNA biosynthesis is an essential core process for protein translation, which is catalyzed by aminoacyl-tRNA synthetases that specifically ligate individual amino acids to their cognate tRNAs to form aminoacyl-tRNA complexes. This pathway plays pivotal roles in maintaining protein synthesis fidelity, amino acid homeostasis, translational reprogramming, and plant abiotic stress tolerance [53,54,55]. In rice, the aspartyl-tRNA synthetase *YLC3* functions to sustain amino acid homeostasis and support normal chloroplast development under low-temperature conditions; *YLC3* mutation results in the accumulation of uncharged tRNA-Asp, repression of protein translation, impaired chloroplast thylakoid structure, and ultimately leaf chlorosis under cold stress [56]. Similarly, the aspartyl-tRNA synthetase IBI1 acts as a perception receptor for the endogenous stress metabolite β-aminobutyric acid (BABA). IBI1 interacts with ABA-inducible VOZ1/VOZ2 TFs to repress ABA-dependent abiotic stress genes and redirect ABA signaling toward callose-associated cell wall defense, thereby coordinating plant immune responses and abiotic stress adaptation [55]. In this study, exogenous trehalose reshaped the amino acid substrate pool involved in aminoacyl-tRNA biosynthesis under heat stress, with most metabolites in this pathway markedly upregulated in the TT group. Consistently, the determination of total amino acid content further confirmed that the TT group possessed significantly higher total amino acid levels than the CK and T groups. These findings demonstrate that trehalose profoundly remodels the amino acid abundance of the aminoacyl-tRNA biosynthesis pathway in heat-stressed tea plants. The increased accumulation of pathway-related amino acids in the TT group provides abundant substrates for protein translation, sustains cellular amino acid homeostasis, and facilitates translational reprogramming, thereby ultimately improving the thermotolerance of tea plants.

In addition, flavone and flavonol biosynthesis is a core defensive pathway for flavonoid production, and these compounds act as major endogenous antioxidants to eliminate excess ROS in plant cells [57,58]. KEGG enrichment analysis revealed that trehalose clearly modulated flavone and flavonol biosynthesis in tea plants under heat stress. Specifically, trehalose treatment increased the contents of flavonoid aglycones (kaempferol and quercetin) while reducing most flavonoid glycosides, with quercetin 3-O-malonylglucoside as an exception. Given that flavonoid aglycones exert stronger antioxidant activity than their glycosylated forms [59,60], this metabolic shift suggests that trehalose is associated with altered flavonoid glycosylation processes, which may further strengthen the antioxidant defense of tea plants under heat stress. We also observed lower accumulation of polyhydroxylated flavonoids (myricetin and laricitrin), which is correlated with changed activities of enzymes encoded by *CsCYP75A1* and *CsCYP75B1*. Such metabolic changes may help plants conserve carbon and energy resources and reallocate metabolites to maintain vital biological processes under heat stress. It has been widely reported that flavonoids accumulate as a typical adaptive response to heat-induced oxidative damage [61]. In our study, the total flavonoid content in the trehalose-treated group (TT) was lower than that in the heat-stressed group (T), but remained higher than the control group. This phenomenon can be explained by our physiological results: trehalose treatment increased the activities of SOD and POD and reduced cellular ROS levels [30,31], thereby effectively alleviating oxidative stress in heat-stressed tea plants. The distinct flavonoid accumulation pattern reflects the dual effects of trehalose. On the one hand, trehalose participates in direct ROS scavenging. Together with elevated antioxidant enzyme activities, it greatly relieves oxidative pressure and reduces plant reliance on flavonoid-based antioxidant defense. On the other hand, trehalose is correlated with altered expression of flavonoid biosynthetic genes including *CsCYP75A1* and *CsCYP75B1*, which in turn modulates flavonoid metabolic profiles. Notably, the reduction in total flavonoids is not caused by suppressed biosynthesis. Collectively, trehalose is linked to coordinated changes in gene expression and metabolite levels, driving comprehensive regulation of flavonoid metabolism in tea plants under heat stress.

As summarized above, exogenous trehalose effectively increases the accumulation of carbohydrates and their conjugates, which act as important osmoprotectants to mitigate heat damage. Meanwhile, trehalose alleviates heat-induced lipid dyshomeostasis and maintains cell membrane integrity. It also modulates multiple amino acid pathways to sustain carbon–nitrogen balance and support normal protein translation. More importantly, trehalose exerted precise regulation on flavonoid metabolism: it elevated the relative abundance of highly antioxidant flavonoid aglycones and inhibited the synthesis of redundant polyhydroxylated flavonoids, thereby optimizing the distribution of carbon and energy resources. These findings demonstrate the distinct metabolic features of heat-stressed tea plants, as well as the specific regulatory patterns of trehalose in this species.

## 4. Materials and Methods

### 4.1. Plant Materials and Heat Stress Treatment

Two-year-old tea plants (*C*. *sinensis* L. cv. Tieguanyin) were cultivated in the tea garden of Ningde Normal University, Ningde, China (26°39′40″ N, 119°35′6″ E). Prior to treatment, the plants were acclimated in an artificial climate incubator under a 12 h light (25 °C)/12 h dark (19 °C) photoperiod with 75% relative humidity for 7 days. After acclimation, the plants were randomly divided into three groups and exposed to heat stress under a 12 h light (38 °C)/12 h dark (29 °C) cycle with 75% relative humidity for 48 h of heat pretreatment. Subsequently, the three groups were subjected to different treatments: (1) CK group: sprayed with distilled water and sampled immediately (0 h of continuous heat stress); (2) T group: sprayed with distilled water and sampled after 24 h of continuous heat stress; (3) TT group: sprayed with 5.0 mM trehalose and sampled after 24 h of continuous heat stress. In our preliminary experiment, we evaluated three trehalose concentrations (2.5, 5.0 and 10 mM) based on leaf phenotypes and multiple physiological indices of tea plants under heat stress. The results showed that 5.0 mM trehalose most effectively alleviated leaf wilting and scorching. After 24 h of heat treatment, this concentration significantly increased the activities of SOD and POD as well as the contents of proline, endogenous trehalose and soluble sugar, and reduced MDA accumulation [30]. For this reason, 5.0 mM was selected as the optimal concentration for the subsequent metabolomic analysis. For each treatment, one bud with the second and third leaves was collected from the top and lateral branches, with three independent biological replicates per group. All samples were immediately frozen in liquid nitrogen and stored at −80 °C until metabolomic analysis.

### 4.2. GC-MS Sample Preparation, Instrumental Analysis, Data Preprocessing, and Statistical Analysis

For GC-MS metabolomic analysis, 60 mg samples were extracted with 600 μL methanol–water (1:1, *v*/*v*, containing 4 μg/mL L-2-chlorophenylalanine) and ground at 60 Hz for 2 min after pre-cooling at −40 °C for 2 min. After ultrasonication in an ice-water bath for 30 min, 150 μL chloroform was added, followed by vortexing, another 30 min ultrasonication, and standing at −40 °C for 30 min. After centrifugation (13,000 rpm, 4 °C, 10 min), 150 μL supernatant was dried and derivatized successively with methoxyamine hydrochloride pyridine solution (15 mg/mL, 37 °C, 60 min) and BSTFA (70 °C, 60 min). Then, 10 μL mixed internal standards (C8–C24) and 20 μL n-hexane were added. After equilibration at room temperature for 30 min, the derivatives were subjected to GC-MS analysis. Quality control (QC) samples were prepared by mixing equal volumes of all sample extracts.

The derivatived samples were analyzed on an Agilent 7890B gas chromatography system coupled with an Agilent 5977A MSD system (Agilent Technologies Inc., Santa Clara, CA, USA). A DB-5MS fused-silica capillary column (30 m × 0.25 mm × 0.25 μm, Agilent J & W Scientific, Folsom, CA, USA) was utilized to separate the derivatives. Helium (>99.999%) was used as the carrier gas at a constant flow rate of 1 mL/min through the column. The injector temperature was maintained at 260 °C. The injection volume was 1 μL in splitless mode. The initial oven temperature was 60 °C, ramped to 125 °C at a rate of 8 °C/min, to 210 °C at a rate of 4 °C/min, to 270 °C at a rate of 5 °C/min, and to 305 °C at a rate of 10 °C/min, and finally, held at 305 °C for 3 min. The temperatures of the MS quadrupole and ion source (electron impact) were set to 150 and 230 °C, respectively. The collision energy was 70 eV. Mass data was acquired in a full-scan mode (*m*/*z* 50–500).

GC/MS raw data (.D) were converted to .abf format using the Analysis Base File Converter, then imported into MS-DIAL for peak detection, identification, MS2Dec deconvolution, characterization, alignment, filtering, and missing value interpolation based on the LUG database. A three-dimensional (3D) data matrix was derived containing sample information, peak name, retention time, retention index, *m*/*z*, and signal intensity. Signal intensities in each sample were normalized using internal standards with RSD > 0.3 after screening, followed by redundancy removal and peak merging. The resulting matrix was imported into R for Principal Component Analysis (PCA) to assess the sample distribution and process stability. Orthogonal Partial Least-Squares-Discriminant Analysis (OPLS-DA) and Partial Least-Squares-Discriminant Analysis (PLS-DA) were utilized to distinguish the metabolites that differ between groups (Supplemental Table S1), with model overfitting evaluated by 7-fold cross-validation and 200 response permutation tests (Supplemental Figure S1). Variable Importance of Projection (VIP) values from OPLS-DA ranked variable contributions, and a two-tailed Student’s *t*-test assessed significance. Differential metabolites were selected with VIP > 1.0 and *p* < 0.05. Each group contained three independent biological replicates, and each was subjected to two technical replicates.

### 4.3. LC-MS Sample Preparation, Instrumental Analysis, Data Preprocessing, and Statistical Analysis

For LC-MS analysis, 60 mg samples were extracted with 600 μL methanol–water (7:3, *v*/*v*) in 1.5 mL centrifuge tubes containing two steel balls. After pre-cooling at −40 °C for 2 min, samples were ground at 60 Hz for 2 min, ultrasonicated in an ice-water bath for 30 min, and stored overnight at −40 °C. Following centrifugation (13,000 rpm, 4 °C, 10 min), 150 μL supernatant was filtered through a 0.22 μm organic membrane and transferred to LC vials for analysis. QC samples were prepared by pooling equal aliquots of all test samples.

LC-MS analysis was performed on an ACQUITY UPLC I-Class Plus system (Waters Corporation, Milford, MA, USA) coupled with a QE Plus high-resolution mass spectrometer (Thermo Fisher Scientific, Waltham, MA, USA) equipped with an electrospray ionization (ESI) source. Separation was carried out on an ACQUITY UPLC HSS T3 column (1.8 μm, 2.1 × 100 mm) using a gradient elution program with water (0.1% formic acid) and acetonitrile (0.1% formic acid) as mobile phases A and B, respectively. The flow rate was 0.35 mL/min, column temperature 45 °C, auto-sampler temperature 4 °C, and injection volume 2 μL. Mass spectra were acquired in positive and negative ion modes over *m*/*z* 100–1200 with a full MS resolution of 70,000 and MS/MS resolution of 17,500.

Raw data were processed using Progenesis QI V2.3 software (Nonlinear, Dynamics, Newcastle, UK) for peak extraction, alignment, and normalization. Compound identification was performed based on an accurate *m*/*z*, secondary fragments, and isotopic distribution by matching against HMDB, Lipidmaps, Metlin, EMDB, PMDB, and an in-house database. Low-quality peaks and compounds with identification scores <36 were removed. Data matrices from positive and negative modes were combined and imported into R for multivariate statistical analysis, including PCA, PLS-DA, and OPLS-DA (Supplemental Table S2). Model validation was performed using 7-fold cross-validation and 200 permutation tests (Supplemental Figure S2). Differential metabolites were identified with VIP > 1.0 and *p* < 0.05. Each group contained three independent biological replicates, and each was subjected to two technical replicates.

### 4.4. Kyoto Encyclopedia of Genes and Genomes (KEGG) Analyses

To elucidate the mechanisms underlying metabolic changes among different groups, KEGG pathway enrichment analysis of differential metabolites was performed using their corresponding KEGG IDs. Hypergeometric tests were applied to identify pathways that were significantly enriched in differentially accumulated metabolites compared with the whole background. The formula is as follows:

P=1−∑i=0m−1MiN−Mn−iNn

where *N* represents the total number of metabolites, *n* represents the number of differential metabolites in *N*, *M* represents the number of metabolites annotated to a specific pathway, and *m* represents the number of differential metabolites annotated to a specific pathway. A threshold of *p*-value ≤ 0.05 was used to define significantly enriched pathways. A smaller *p*-value indicates a more significant difference in the corresponding metabolic pathway.

### 4.5. Transcriptome Sequencing and Bioinformatics Analysis

Total RNA was extracted with the mirVana miRNA Isolation Kit, and qualified RNA samples were used for rRNA removal and library construction using the VAHTS Universal V6 RNA-seq Library Prep Kit [62]. All libraries were subjected to Illumina sequencing. Raw sequencing data were filtered by Trimmomatic and fastp to obtain clean reads [62,63], which were subsequently mapped to the tea plant reference genome via Hisat2 [64]. Gene expression was quantified using HTSeq and normalized as FPKM [65,66]. DEGs were identified by DESeq with the thresholds of |log_2_FC| > 1 and *p* < 0.05 [67]. KEGG pathway enrichment of DEGs was performed using the ClusterProfiler package, and pathways with *p* < 0.05 were regarded as significantly enriched [68].

### 4.6. Determination of Amino Acid Content in Tea Leaves

The total amino acid of tea leaves was determined using a total amino acid content assay kit (Grace Biotechnology, Suzhou, China) following the manufacturer’s instructions. In brief, the content of total amino acids was determined by the ninhydrin color developing method [69]. Around 0.1 g leaf samples was homogenized in 1 mL distilled water. The homogenate was heated in a boiling water bath for 15 min, cooled, and centrifuged at 8000 *g* for 10 min at 25 °C. The supernatant was collected for analysis.

After mixing with the kit reagents, the sample was heated in a boiling water bath for 15 min. Absorbance was detected at 570 nm within 30 min, and the amino acid content was calculated according to the standard curve. Each assay was performed in triplicate.

### 4.7. Determination of Flavonoid Content in Tea Leaves

The flavonoid content of tea leaves was determined using a commercial assay kit (Comin Biotechnology, Suzhou, China) following the manufacturer’s protocols. Briefly, approximately 0.1 g fresh tea leaves was ground and mixed with 2 mL of 60% ethanol. The mixture was subjected to shaking extraction at 60 °C for 2 h, then centrifuged at 10,000 *g* and 25 °C for 10 min. The resulting supernatant was collected for subsequent measurement.

Distilled water was used for blank calibration. Samples and blank controls were mixed sequentially with Kit Reagent following the specified volume and incubation conditions. After reaction, the absorbance was measured at 510 nm. The flavonoid content was calculated using the standard curve equation given in the kit instructions. All assays were performed in triplicate.

### 4.8. Data Analysis

The statistical analysis was performed using one-way ANOVA with IBM SPSS Statistics 27 (SPSS Inc., Chicago, IL, USA). Differences were considered statistically significant at *p* < 0.05. Bar graphs were generated using GraphPad Prism 8.0 (GraphPad Software, San Diego, CA, USA).

## 5. Conclusions and Future Prospects

**Figure 9:**
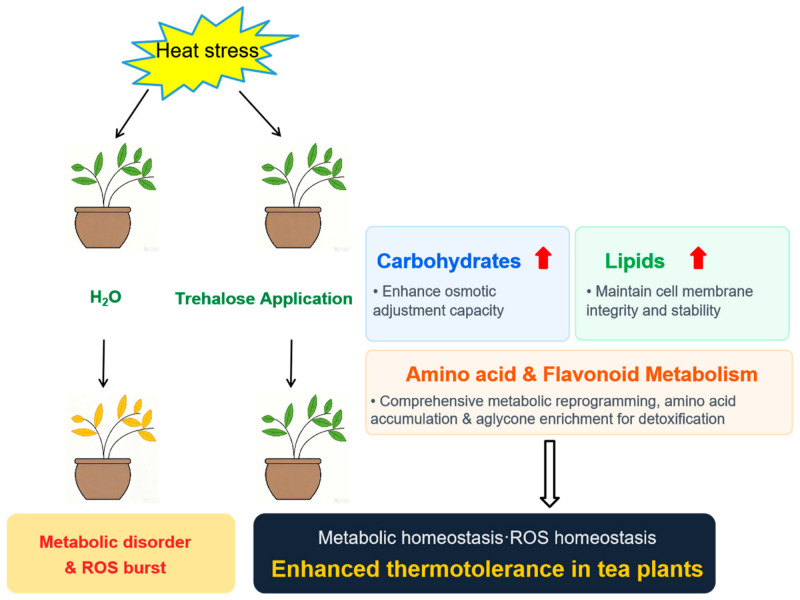
Proposed model illustrating the mechanism by which exogenous trehalose enhances thermotolerance in tea plants under heat stress. Heat stress triggers metabolic disorder and ROS burst in tea plants, leading to leaf senescence (yellowing). Exogenous trehalose application regulates multiple metabolic pathways to alleviate heat-induced damage: (1) it promotes carbohydrate accumulation to enhance osmotic adjustment; (2) it restores lipid homeostasis to maintain cell membrane integrity; and (3) it reshapes amino acid and flavonoid metabolism, including regulation of alanine-aspartate-glutamate metabolism, arginine biosynthesis, aminoacyl-tRNA biosynthesis, and flavone/flavonol biosynthesis. Collectively, these trehalose-mediated regulations restore metabolic and ROS homeostasis, thereby improving the heat stress tolerance of tea plants.

In summary, exogenous trehalose modulates the metabolic landscape of tea plants under heat stress primarily by promoting the accumulation of carbohydrates and carbohydrate conjugates, and lipids and lipid-like molecules. Trehalose significantly regulates key metabolic pathways including alanine, aspartate and glutamate metabolism, arginine biosynthesis, and aminoacyl-tRNA biosynthesis. It increases the abundance of functional amino acids, maintains carbon–nitrogen homeostasis, and provides sufficient substrates for accurate protein translation, thereby enhancing osmotic adjustment and basal stress tolerance of tea plants. Meanwhile, trehalose regulates flavonoid modification by promoting the accumulation of antioxidant-active aglycones (kaempferol, quercetin) and inhibiting most of their glycoside forms; notably, it reduces the total flavonoid content compared to the heat-stressed T group. This dynamic change indicated that trehalose effectively alleviates heat stress damage, thereby reducing the tea plants’ need for excessive flavonoid synthesis and altering metabolic resources to core stress-defensive processes. The coordinated regulation of these metabolic processes enables tea plants to maintain metabolic homeostasis under high-temperature stress, thus improving their heat stress tolerance (Figure 9).

For future research, three targeted research directions are proposed based on the above findings. First, further investigations should focus on the upstream signaling networks that govern the metabolic reprogramming of carbohydrates, lipids, amino acids and flavonoids triggered by trehalose. Combined with targeted metabolomics, transcriptomics and gene editing approaches, the core genes and pivotal metabolites involved in alanine–aspartate–glutamate metabolism, arginine biosynthesis and flavonoid modification can be functionally verified. Second, the screened differential metabolites, especially kaempferol, quercetin and characteristic amino acids, can be developed as physiological biomarkers for rapid identification of heat-tolerant tea germplasms. The key genes associated with trehalose metabolism and flavonoid modification can be exploited to cultivate new heat-resistant tea varieties via molecular breeding strategies. Third, systematic field trials need to be carried out to optimize the optimal concentration, spraying frequency and application period of exogenous trehalose for tea plantations. The synergistic effects between trehalose and other eco-friendly plant growth regulators can be evaluated. In particular, long-term monitoring of flavor-related amino acids and volatile compounds is required to comprehensively assess the effects of trehalose on tea yield and sensory quality under natural high-temperature environments.

Collectively, this study clarifies the metabolic regulatory mechanism by which trehalose mitigates heat stress damage in tea plants, and provides a new theoretical basis and technical reference for the application of trehalose in tea plant production to cope with high-temperature stress.

## Data Availability

All data presented in this study are provided either in the manuscript or Supplementary Materials.

## Supplementary Materials

The following supporting information can be downloaded at: https://www.mdpi.com/article/10.3390/plants15131938/s1. Figure S1: 200 response permutation tests of OPLS-DA models for GC-MS metabolomic analysis: (A) T vs. CK; (B) TT vs. CK; (C) TT vs. T. R^2^, where Q^2^ represents parameters of response permutation testing, used to assess whether the model is overfitted; Figure S2: 200 response permutation tests of OPLS-DA models for LC-MS metabolomic analysis: (A) T vs. CK; (B) TT vs. CK; (C) TT vs. T. R^2^, where Q^2^ represents parameters of response permutation testing, used to assess whether the model is overfitted; Table S1: Parameters of OPLS-DA and PLS-DA models for GC-MS metabolomic analysis (T vs. CK, TT vs. CK, TT vs. T); Table S2: Parameters of OPLS-DA and PLS-DA models for LC-MS metabolomic analysis (T vs. CK, TT vs. CK, TT vs. T); Table S3: Identification of 163 differential volatile metabolites (DVMs) in CK, T, and TT groups; Table S4: Identification of 1619 differential non-volatile metabolites (DNMs) in CK, T, and TT groups.
