# Supplementary figures and images for: Non-Targeted Metabolomic Analyses Provide Insights into Exogenous Trehalose-Mediated Heat Stress Tolerance in Tea Plants (Camellia sinensis L.)
Source: Plants (Basel). 2026 Jun 23;15(13):1938. doi: 10.3390/plants15131938 (PMC13364077; doi:10.3390/plants15131938)

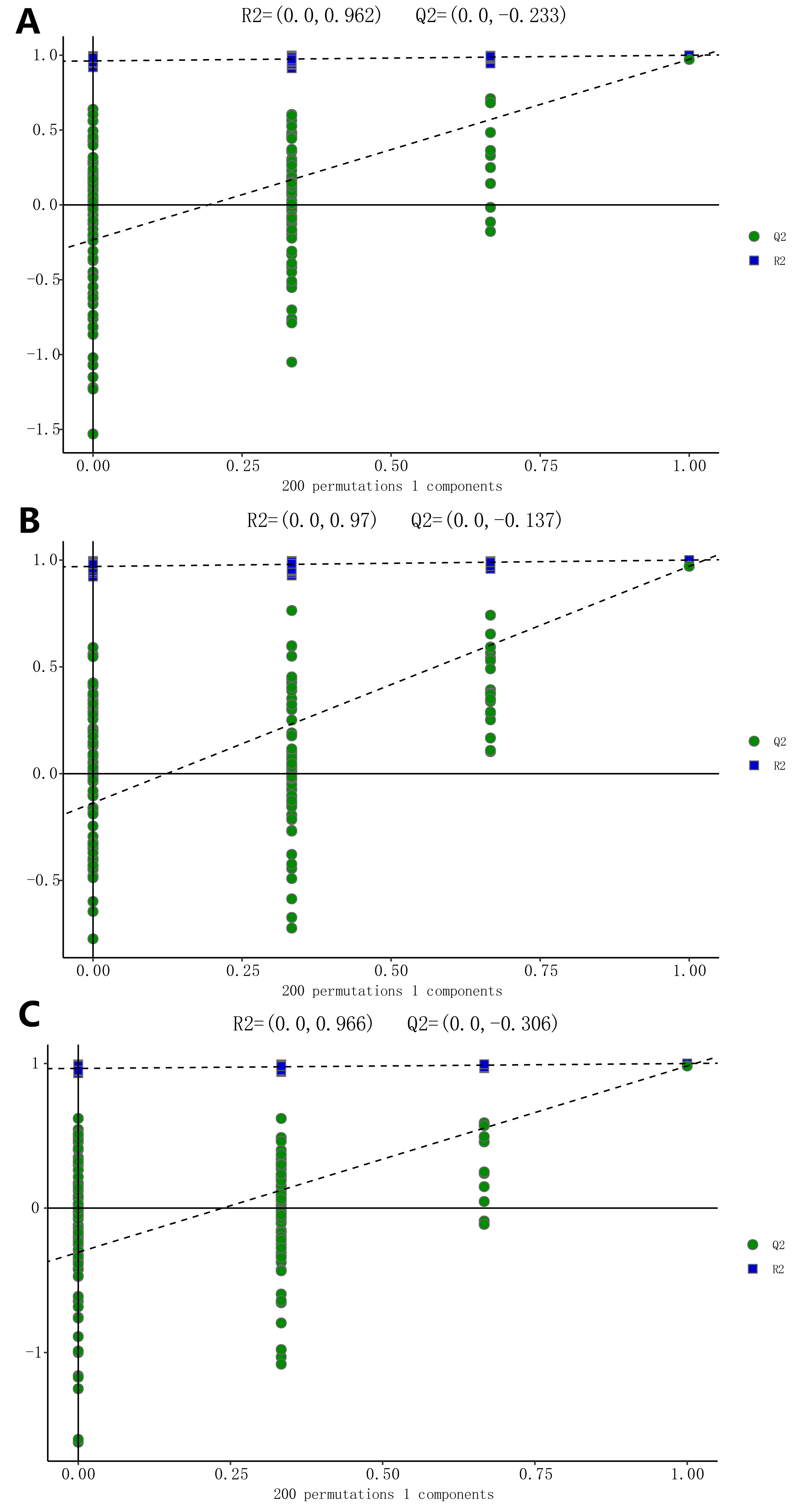

Supplement: Supplementary file 1 [file plants-15-01938-s001.zip › Figure S1.tif]

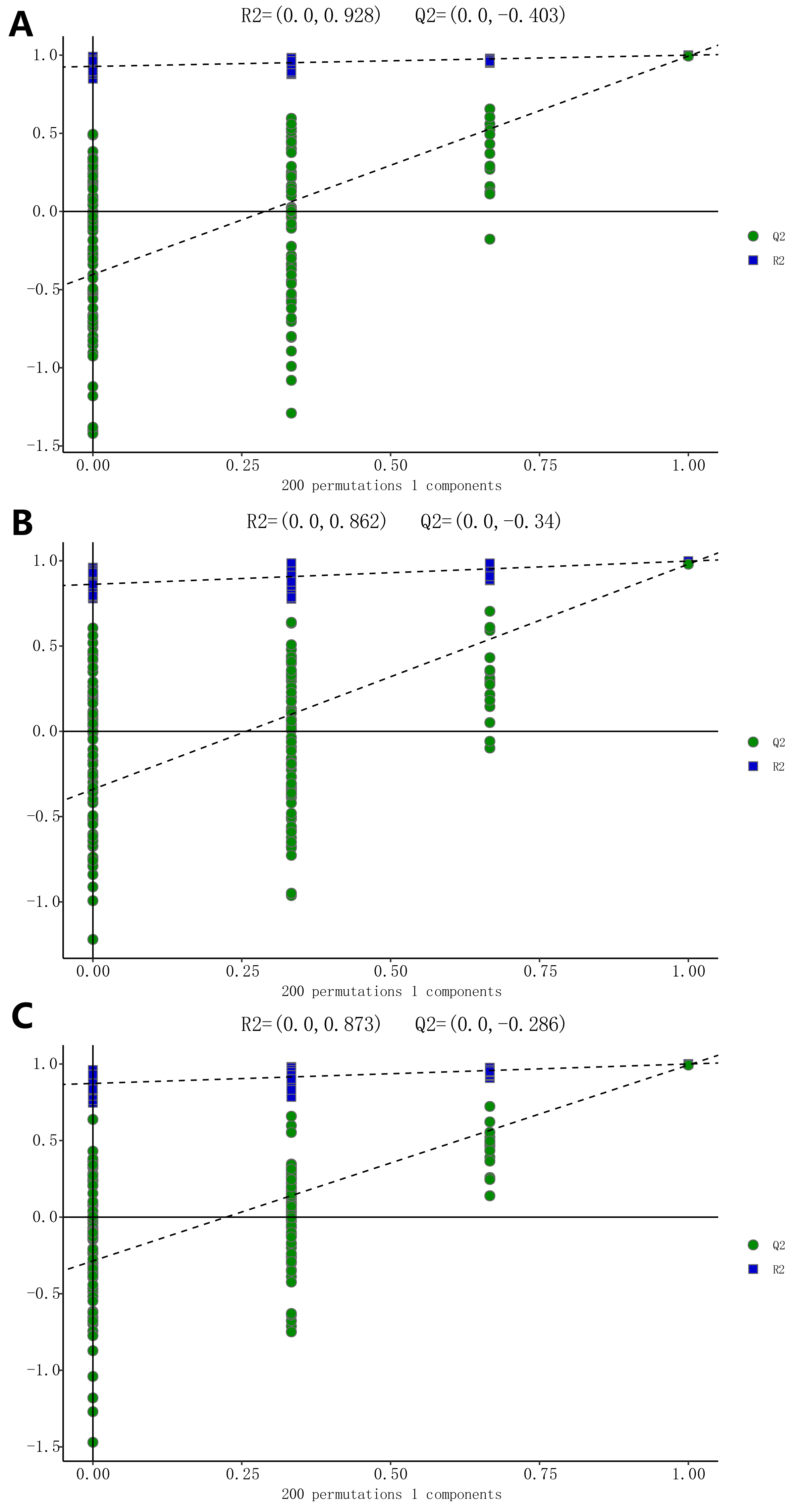

Supplement: Supplementary file 1 [file plants-15-01938-s001.zip › Figure S2.tif]
